# *Rs1h*^*−/y*^ exon 3-del rat model of X-linked retinoschisis with early onset and rapid phenotype is rescued by *RS1* supplementation

**DOI:** 10.1038/s41434-021-00290-6

**Published:** 2021-09-22

**Authors:** Yong Zeng, Haohua Qian, Maria Mercedes Campos, Yichao Li, Camasamudram Vijayasarathy, Paul A. Sieving

**Affiliations:** 1 National Eye Institute, National Institutes of Health, Bethesda, MD USA; 2 Department of Ophthalmology, University of California Davis, Sacramento, CA USA; 3 Present Address: Center for Ocular Regenerative Therapy, Department of Ophthalmology and Vision Science, University of California Davis, Sacramento, CA USA

**Keywords:** Visual system, CNS cancer

## Abstract

Animal models of X-linked juvenile retinoschisis (XLRS) are valuable tools for understanding basic biochemical function of retinoschisin (RS1) protein and to investigate outcomes of preclinical efficacy and toxicity studies. In order to work with an eye larger than mouse, we generated and characterized an *Rs1h*^*−/y*^ knockout rat model created by removing exon 3. This rat model expresses no normal RS1 protein. The model shares features of an early onset and more severe phenotype of human XLRS. The morphologic pathology includes schisis cavities at postnatal day 15 (p15), photoreceptors that are misplaced into the subretinal space and OPL, and a reduction of photoreceptor cell numbers by p21. By 6 mo age only 1–3 rows of photoreceptors nuclei remain, and the inner/outer segment layers and the OPL shows major changes. Electroretinogram recordings show functional loss with considerable reduction of both the a-wave and b-wave by p28, indicating early age loss and dysfunction of photoreceptors. The ratio of b-/a-wave amplitudes indicates impaired synaptic transmission to bipolar cells in addition. Supplementing the *Rs1h*^−/y^ exon3-del retina with normal human RS1 protein using AAV8-*RS1* delivery improved the retinal structure. This *Rs1h*^−/y^ rat model provides a further tool to explore underlying mechanisms of XLRS pathology and to evaluate therapeutic intervention for the XLRS condition.

## Introduction

Animal models of human diseases have been widely used to study features of human monogenic diseases and to explore treatment strategies. Several *Rs1h*^*−/y*^ knockout (KO) mouse models have been generated which mimic the human pathology in X-linked juvenile retinoschisis (XLRS) patient [1–4]. All of these genetically modified mice fail to express normal retinoschisin protein, and they display structural and functional retinal phenotypes in the spectrum of XLRS patients. Histologically, retina laminar structures are disorganized with photoreceptor cells mis-localized in early to mid-stage, and the inner nuclear layer has schisis structural abnormalities. Synaptic transmission in the outer plexiform layer, as is also involved in the human XLRS disease [5, 6], and photoreceptor cells are degenerated in mid-late stages. Functionally, these mice show the characteristic reduction of dark-adapted ERG b-wave amplitudes and a reduction of the b-/a-wave amplitude ratio in many XLRS patients [1]. There are currently no effective treatments for XLRS disease, and efforts are underway to ameliorate the condition using clinical AAV-mediated gene therapy (ClinicalTrials.gov.NCT 02317887 and NCT 02416622) [1, 5, 7–12].

The small eye of *Rs1h*-mutant mice presents a challenge to explore treatments. Injecting therapeutic agents into the small eye risks ocular damage and a rapid increase of intraocular pressure (IOP) [13] which may confound interpretation of results in pre-clinical studies [7]. In previous pre-clinical safety and toxicity study for a XLRS gene therapy trial [14], we used rabbit for some of the studies. It would be useful to have a larger animal disease model that represents XLRS human disease. Here we report results of creating and characterizing an *Rs1h*^−/y^ exon3-del rat model using CRISPR/Cas 9 techniques [15–17].

## Materials and methods

Experiments were conducted in accordance with the ARVO Statement for the Use of Animals in Ophthalmic and Vision Research and with protocols approved by the National Eye Institute Animal Care and Use Committee. Normal Long Evans rats were purchased from Charles River Laboratory (Wilmington, MA) and housed under fluorescent lights in a 12-h light–dark cycle.

### Generation of *Rs1h*^−/y^ exon3-del rat model

The *Rs1h*^*−/y*^ rat was generated using CRISPR/Cas9-mediated gene editing system [15–19]. Exon 3 (106 base pair) of rat *Rs1h* (GenBank accession number: NM_001104643.1; Ensembl: ENSRNOG00000030434) was targeted with two guide RNAs (gRNA) located in intron 2 and intron 3 to introduce two DNA breaks with Cas 9 endonuclease. gRNAs and Cas9 nuclease mRNA were transcribed in vitro and were injected into the pronuclei of fertilized eggs of Long Evans rat and implanted into surrogate mothers to obtain founders. Two founders were identified by PCR genotyping and confirmed by DNA sequencing analysis. They were then bred with wildtype Long Evans to produce generation 1 (F1), and correct transmission of the mutation was confirmed by PCR genotyping and by DNA sequencing analysis. Hemizygous male (*Rs1h*^−/y^) F1 and heterozygous female (*Rs1h*^−/+^) F1 was bred with wildtype Long Evans to establish rat colonies. The *Rs1h*^*−/y*^ rat model was generated with assistance from Cyagen Biosciences Inc. (Santa Clara, CA, USA). Ten potential off-target sites were predicted on chromosomes 2, 3 (two sites), 4, 7, 9, 10, 14, 16, and X. These were amplified with PCR method, the absence of any of these potential mutations was verified using DNA sequencing analysis of PCR products in all F1 rats.

### Absence of normal Rs1h protein in *Rs1h*^−/y^ rat model

#### Western blot analysis

Rats were euthanatized by carbon dioxide followed by bilateral pneumothorax, and retinas were dissected. Retinal extract was obtained by lysing in RIPA buffer (25 mM Tris-HCl [pH 7.6], 150 mM NaCl, 1% NP40, 1% sodium deoxycholate, 0.1% SDS) containing protease inhibitor cocktail (Thermo Scientific, Rockford, IL, USA). Protein concentrations were determined by the Pierce Bicinchoninic acid (BCA) Protein Assay Kit (Thermo Scientific, Rockford, IL, USA) with bovine serum albumin as the standard. Thirty micrograms of total protein from each sample was separated on duplicate SDS-PAGE gels (Thermo Fisher/Life Technologies, Carlsbad, CA, USA), and transferred onto separated polyvinylidene difluoride membrane (Bio-Rad Laboratories, Hercules, CA, USA), one for Western blot analysis, and the second was stained with Ponceau S solution (Sigma, St. Louis, MO, USA) to demonstrate the similar loading of retinal extract and transferring efficiency across all samples. The Western blot membrane was incubated overnight with a rabbit polyclonal RS1 antibody against the N terminus of retinoschisin (amino acid residues 24–37), and then was rinsed in 0.1% Tween-20 in sodium phosphate buffer (PBS) followed by incubation with IRDye 800CW conjugated goat anti-rabbit IgG (Cat# 926-32211; LI-COR Biosciences, Lincoln, NE, USA). Blot was scanned on LI-COR Odyssey Infrared Imaging System (Model 9120; LI-COR Biosciences, Lincoln, NE, USA).

#### Immunofluorescent assays

Animals were euthanized, and the eye globe was enucleated with the third eyelid retained. This was fixed in 4% paraformaldehyde (PFA) in sodium phosphate buffer (PBS) on ice for 30 min, and the cornea and lens were removed to make eyecup. The eyecups were returned to fixation buffer for 1.5 h and processed for cryosectioning. The 10 μm sagittal cryosections were cut for immunofluorescent assay [13]. Briefly, retinal sections were rinsed in washing buffer (0.1% Triton X-100 in PBS) and preincubated with PBS containing 20% normal goat serum (Sigma, Steinheim, Germany) and 0.5% Triton X-100 at room temperature (RT) for 2 h to block nonspecific antibody binding. The retinal sections were incubated overnight at 4 °C with a rabbit polyclonal antibody against the N-terminus of retinoschisin (amino acid residues 24–37 (1:1000, customized antibody, Thermo Fisher Scientific, Waltham, MA, USA) and/or a monoclonal antibody against the PKCα (1:500, Santa Cruz, Dallas, TX, USA) in PBS buffer containing 5% normal goat serum, after rinsing with washing buffer, sections were incubated with an secondary antibody conjugated to red-fluorescent Alexa Fluor 568 dye or green-fluorescent Alexa Fluor 488 dye (Thermo Fisher Scientific, Waltham, MA, USA) for 1 hour at room temperature. The nuclei were stained with 40,60—diamidino-2-phenylindole (DAPI; Thermo Fisher Scientific, Waltham, MA, USA) in washing buffer, and sections were mounted with Fluorogel (Electron Microscopy Sciences, Hetfield, PA, USA). Retinal images were obtained and processed with a Nikon C2 confocal microscope with Advanced Element software (Nikon, Tokyo, Japan). Image analysis was performed using image-editing software (Photoshop CS6; Adobe Systems, Inc., San Jose, CA, USA).

### Optical coherence tomography (OCT)

#### OCT image collection

Retinal OCT optical images were obtained with the Envisu R2200 SD-OCT (Bioptigen, Durham, NC, USA). Animals were anesthetized by intraperitoneal injection of ketamine (100 mg/kg) and xylazine (10 mg/kg). Pupils were dilated with tropicamide and phenylephrine. Artificial tears (Alcon Laboratories, Inc., Fort Worth, TX, USA) were used throughout the procedure to maintain corneal clarity. For rats at p8-p10 the eyelids are not yet open, and to scan at this age, a knife slit was made in the junction between the upper and lower lids of anaesthetized, and eyelids were opened with a forceps. Radial volume scans consisting of 4 B-scans (1000 A-scans per B-scan) were collected at 45 angular intervals and were each an average of five frames. Rectangular volume scans with 100 B-scans (1000 A-scans per B-scan) were collected, across a 2.6 × 2.6-mm area centered on the ON head. To examine the peripheral retina, the probe angle to the eye was oriented for maximum viewing of the retina. Cavity size, inner retina thickness, outer retina thickness, and outer retina reflective band (ORRB) number and morphology were identified to evaluate the retinal structure. For natural history study, we monitored *Rs1h*^*−/y*^ male rats and WT littermates at six different ages, including ages of postnatal days 15 (p15), p21, p28, and 2, 3 and 6 mo. Measurements at each time point included at least 5 *Rs1h*^*−/y*^ rats and 3 WT rats.

#### OCT layer thickness measurements

Retinal layer thickness was measured from radial scan OCT images (2. 6 mm at 1000 A-scan x 4 B-scan x 100) using a custom made Matlab program. Briefly, OCT images of 650 µm to 1170 µm away from optic nerve head (i.e., 51–250 pixels from image edge) were analyzed. Each image was binned every 10 pixels, and the lines of inner limiting membrane (ILM), external board of inner plexiform layer (IPL), and Bruch’s membrane (BM) from both WT and *Rs1h*^−/y^ OCT images were marked and user verified. Thickness from ILM to IPL was measured for evaluating inner retinal thickness. Retinal distal portion from external board line of IPL to BM was measured because outer retina from ONL to RPE cannot be measured directly due to the indistinctive boarder lines for INL, OPL, and ONL in *Rs1h*^*−/y*^ rat. As the INL cell counts in *Rs1h*^*−/y*^ were the same as WT at all ages checked, we postulated that the INL thickness of both WT and *Rs1h*^*−/y*^ in OCT images should also be similar. Therefore, INL thickness of WT retina was measured, and the values were used to calculate outer retina thickness (OPL to RPE) for both WT and *Rs1h*^*−/y*^ mice.

#### Histology

Eyes were enucleated from euthanatized animals and fixed in 10% formalin for histology examination. After dehydration, the eye globes were embedded in rapid polymerizing methyl methacrylate. Sagittal semithin sections were cut through the center of eye, and these including ON head were stained with hematoxylin and eosin (H&E). Retinal images were collected using an Axio Scan Z.1. Zeiss microscope (Carl Zeiss Microscopy, Jena, Germany) and analyzed using image-editing software (Photoshop CS6; Adobe Systems, Inc., San Jose, CA, USA). The ONL and INL cells of inferior and superior retina were counted separately between 500 and 1000 μm from the optic nerve (ON) using an automated method with ImageJ software (http://imagej.nih.gov/ij; provided in the public domain by the National Institutes of Health, Bethesda, MD, USA). The cell counts per 100 μm length of retina was used to evaluate the ONL and INL cell number. For the natural history study, eye globes from *Rs1h*^*−/y*^ male rats and WT littermates were collected at p15, p21, p28, and 2, 3 and 6 mo. H&E stained sagittal semithin sections including the ON head were evaluated. For heterozygous females, retinal morphology was evaluated at 1, 3, 6, and 12 mo. Each group included at least 5 *Rs1h*^*−/y*^ rats or 3 *Rs1h*^*−/+*^ rats and 2 WT rats.

#### Electroretinography (ERG)

Full-field ERGs were recorded from 13 male *Rs1h*^*−/y*^ and 7 male WT Long Evans rats at p27-p28 using an Espion E2 system (Diagnosys, Lowell, MA). Animals were dark adapted overnight and then anesthetized with intraperitoneal ketamine (100 mg/kg) and xylazine (6 mg/kg). The pupils were dilated with topical 0.5% tropicamide and 2.5% phenylephrine HCl. Rats were placed on a heating pad to maintain body temperature. ERGs were recorded with gold loop wires placed on the cornea with a drop of methylcellulose after application of 1% proparacaine topical anesthetic. A gold wire was placed in the mouth as the differential electrode, and a ground wire was attached to the tail. Dark-adapted ERG responses were elicited with flash intensities from –3.8 to 0.7 log cd.s/m^2^ in 0.5-log steps. Ganzfeld (full-field) stimuli were delivered by a ColorDome sphere (Diagnosys, Lowell, MA), with interstimulus intervals of 3–60 s depending on stimulus intensity. Responses were frequency filtered using a 0.3-Hz–300 Hz bandpass and a 60-Hz line-frequency filter. A-waves were measured from the pre-stimulus baseline to the initial negative trough. B-waves were measured from the baseline or from the a-wave trough when present. Intensity-response relations of the a- and b-waves elicited by low intensity flash (–3.8 to −0.7 log cd.s/m^2^) are primarily from the rod photoreceptor circuits. They were fitted (GraphPad Prism V9; GraphPad Software Inc.) by the Naka-Rushton equation:

$$R = R_{\max }\frac{{I^n}}{{I^n + K^n}}$$

where R_max_ is the maximal response amplitude, I is the flash intensity, n is the Hill coefficient, and K is the half-saturation constant.

#### Mutant *Rs1h* mRNA detection and confirmation

Reverse transcript (RT)-PCR was performed to detect the message RNA (mRNA) of *Rs1h*. Retinas were collected, and the total RNA was extracted and purified using RNAzol RT (Sigma, St. Louis, MO, USA). The 2 μg of total RNA was used to synthesize first strand cDNA with random primers and M-MLV reverse transcriptase (Invitrogen, Eugene, OR, USA). *Rs1h* transcript was amplified using cDNA templates, a pair of *Rs1h* gene specific primers and platinum Taq polymerase (Invitrogen, Eugene, OR, USA). The sequence of the forward primer was 5ʹ-AGGCTTCTTCTTATTGCTTCTCTT-3ʹ, the sequence of the reverse primer was 5ʹ-AGTCGGATGAAGCGGGAAATAATG-3ʹ. PCR tests were performed with 22 cycles, a number empirically determined within the linear range of PCR analysis (data not shown). RT-PCR products were visualized in Flashing gel system (Lonza, Alpharetta, GA, USA), and images were taken using FlushGel camera (Lonza, GA, USA). The Density of RT-PCR bands was analyzed using Image J 1.48 v (http://imagej.nih.gov/ij; provided in the public domain by the National Institutes of Health, Bethesda, MD, USA). RT-PCT products were purified with QIAquick PCR Purification Kit (Qiagen, Germantown, MD, USA) and the RT-PCR products were sequenced and compared with the expected target DNA sequence.

#### AAV delivery of human *RS1* gene augmentation

*Rs1h*^*−/y*^ male rats were administrated 2e10 viral genome per eye of scAAV8-hRS/IRBP-*hRS*, the vector used in our XLRS clinical trial [9], at p7-p8 following the procedure as described previously [7, 12]. One eye was administrated vector by intravitreal injection, and contralateral eye was an untreated control. Twelve weeks post injection (PI), OCT images were collected, and eyes were enucleated and prepared for cryo-sectioning. The vector expression of *RS1* and retinal morphology was assessed as described previously [7]. Immunofluorescent images were captured using the Nikon C2 confocal microscope as described above.

## Results

### Generation of *Rs1h*^*−/y*^ Exon-3 deletion XLRS rat model

**Fig. 1:**
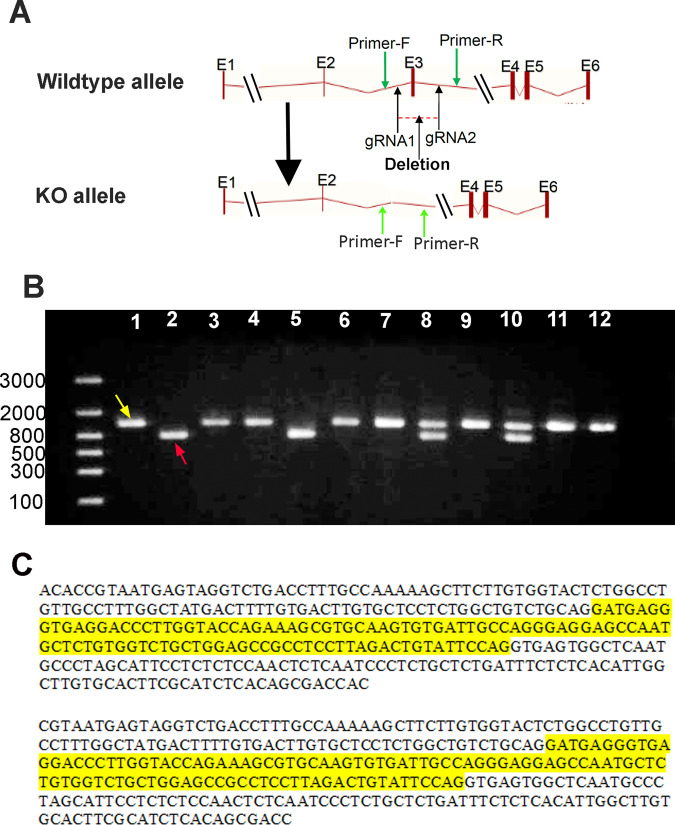
*Rs1h*^−/y^ rat model generation and genotyping strategy. CRISPR/Case9 genome editing system with two guide RNAs was used to generate the knockout rat model. **A** top depicts the wildtype allele of *Rs1h* with the locations of gRNA1 within intron 2 and gRNA2 within intron 3. The exon 3 deletion of *Rs1h* knockout allele was shown in (**A**) bottom diagram. Forward primer of genotyping located at the upstream of gRNA1 and reverse primer located at the downstream of gRNA2. One band of 1051 bp was shown in WT (1B, lane 1, 3, 4, 6, 7, 9, 11, and 12), one band of 750 bp was shown in *Rs1h*^−/y^ (1B, lane 2 and 5), and both bands of 1051 bp and 750 bp were shown in heterozygous female (**B**, 8, and 10) from all the samples used in this study. DNA sequencing of PCR products revealed that a 316 base deletion encompassing exon 3 and partial intron 2 and intron 3 in one founder (**C**, top sequences), and another founder had a 310 base deletion of exon 3 highlighted in yellow, partial intron 2 and intron 3 (**C**, lower sequences) “(color figure online)”.

Figure 1A depicts the strategy of generating *Rs1h*^*−/y*^ rat with CRISPR/Cas9 system using guide RNA1 (gRNA1) located within intron 2 and guide RNA2 (gRNA2) within intron 3. These two gRNAs lead Cas 9 endonuclease to the target region and make DNA breaks followed by non-homologs end-joining which excises exon 3. Two founders were generated with exon-3 deletions (Fig. 1C), and colonies were established. As they had essentially the same phenotype, results were pooled. Genotypes were determined using forward primers upstream of gRNA1 and a reverse primer downstream of gRNA2, and the targeted region encompassing exon 3 was amplified. This gives a 1051 base pair (bp) fragment for the WT allele (Fig. 1B, large band, yellow arrow) and a smaller 750 bp fragment for the *Rs1h* knockout allele (Fig. 1B, red arrow). DNA sequencing confirmed that the deletion encompassed exon 3 plus partial sequence of adjoining intron 2 and intron 3 (Fig. 3C, yellow highlight). The loss of exon 3 causes an *Rs1h* frameshift downstream which results in multiple premature termination codons (PTCs).

### Characterization of *Rs1h*^*−/y*^ rat

#### Loss of normal Rs1h protein in *Rs1h*^*−/y*^

**Fig. 2:**
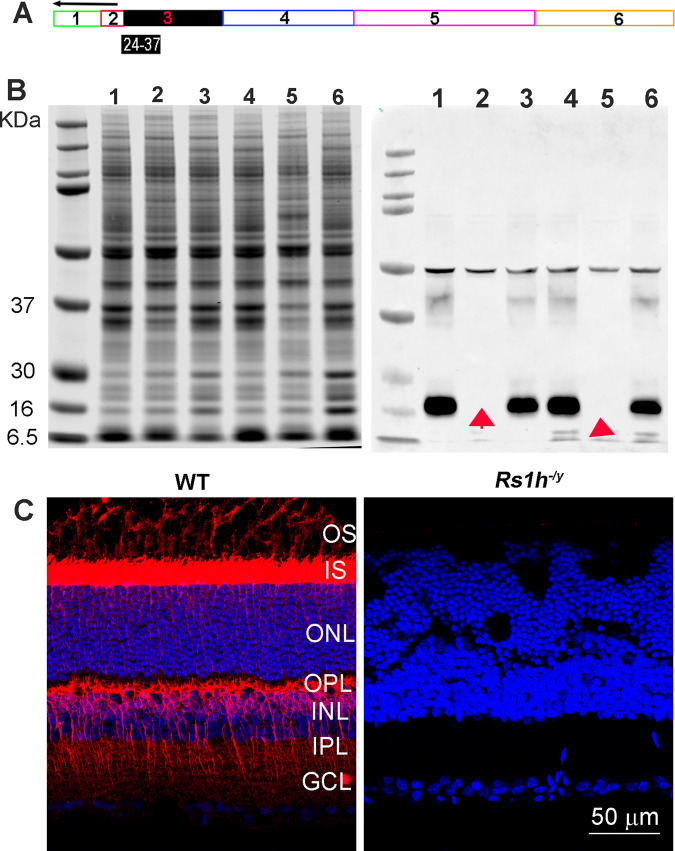
Absence of normal Rs1h protein in retinal extract and retinal section. **A** Diagram of Rs1h protein translated from *Rs1h* exon 6. The solid black rectangular represents the 24–37 amino acid used to generate RS1 antibody; the black arrow indicates the signal sequence that is cleaved in mature Rs1h protein. Consequently this antibody cannot identify truncated protein translated when exon 3 is excised from mRNA. **B** left, Western blot of retinal extract. The 24 KDa band corresponds to Rs1h protein in lane 1 and 4 of WT retinas and also lane 3 and 6 of heterozygous retinas, but it is absent in lane 2 and 5 of exon 3 knock out retinas (right panel, red arrowhead). **B** left, duplicate membrane of right panel is stained with Ponceau S solution to demonstrate equal protein loading and the same protein transfer efficiency across all retinal samples. **C** Shows RS1 antibody labeled immunofluorescent images of WT retina (left) and *Rs1h*^−/y^ retina (right). Multiple WT retinal layers show robust Rs1h expression, but not in *Rs1h*^−/y^ retina (right). IS inner segment, OPL outer plexiform layer, INL inner nuclear layer, IPL inner plexiform layer. Scale bar: 50 μm “(color figure online)”.

The absence of normal Rs1h protein was confirmed by Western blot and immunohistochemistry (IHC). Total retinal protein extracts (Fig. 2B left) were analyzed using a polyclonal RS1 antibody against amino acid 24-37 that are translated from exons 2 and 3 (Fig. 2A). This showed a 24 kD band corresponding to the WT normal size Rs1h protein (Fig. 2B, right panel, lane 1 and 4) and also for the *Rs1h*^*−/+*^ heterozygote (Fig. 2B, right panel, lane 3 and 6), but this was absent for *Rs1h*^*−/y*^ males (Fig. 2B, right pane, lane 2 and 5, red arrows). IHC analysis of retinal sections with this antibody detected no Rs1h protein expression in *Rs1h*^−/y^ (Fig. 2C right), whereas WT retina showed retinoschisin expression in multiple retinal layers, including the inner segment layer (IS), outer plexiform layer (OPL) and inner nuclear layer (INL) (Fig. 2C, left) as expected for WT mouse [20, 21].

#### Morphological abnormalities and natural history study

**Fig. 3:**
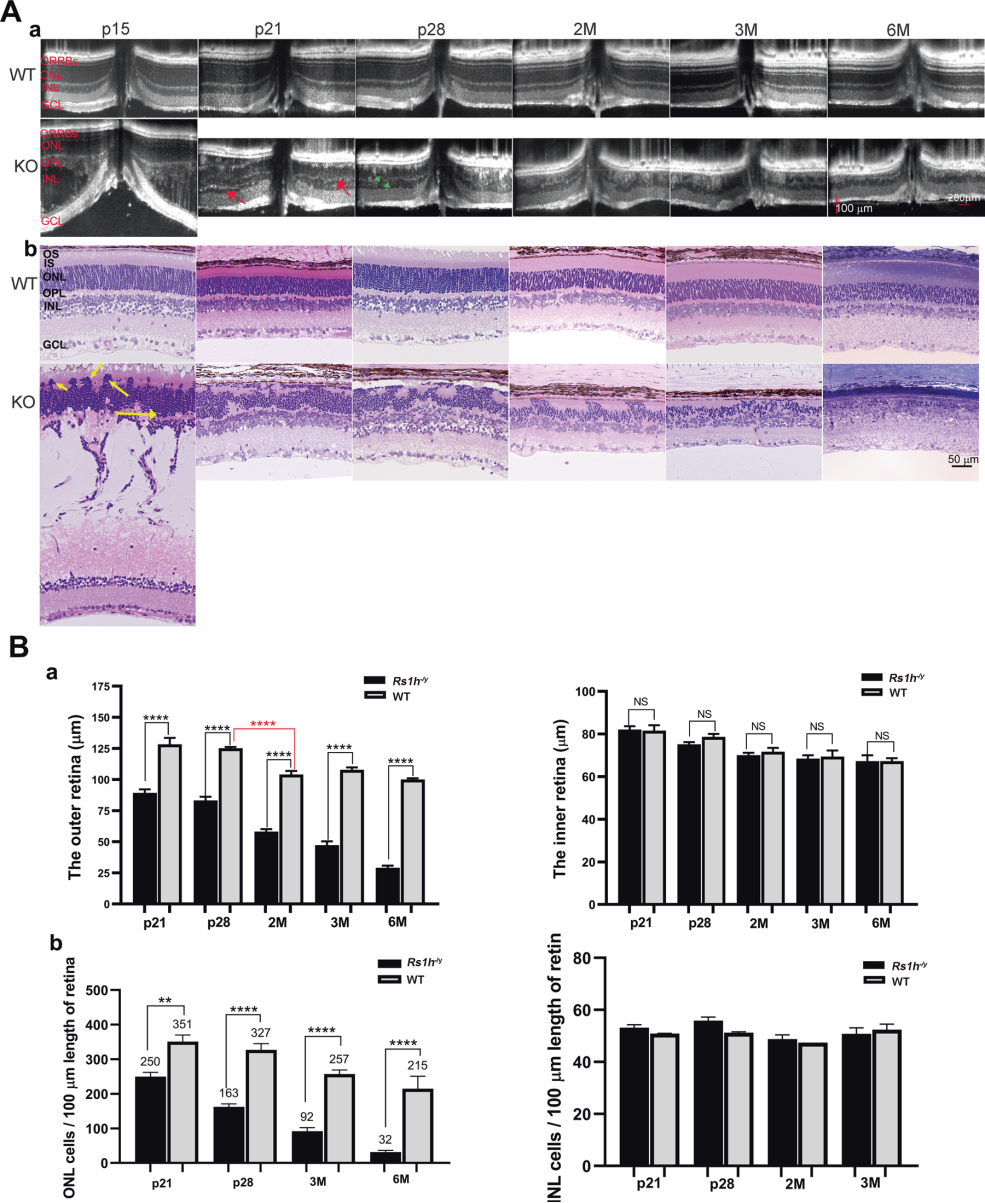
Phenotype features of *Rs1h*^−/y^ rat model. OCT retinal images (Fig. 3Aa) and semithin histology sections (Fig. 3Ab) show retinal morphology changes at six ages (p15, p21, p28, 2 mo, 3 mo and 6 mo). Fig. 3Aa bottom, retinal morphology of *Rs1h*^−/y^ rat shows a giant cavity by age p15, which narrowed to a crevice in INL by p21 (red arrows), and nearly disappeared by p28 (green arrows) (normal retinal morphology of WT, Fig. 3Aa top). The ONL thinned rapidly with age, retinal laminar structure was disrupted, and the OPL to OS margin became indistinctive. The INL had irregular boarders, but INL thickness remained relatively normal. *Rs1h*^−/y^ retinal histology at corresponding ages (Fig. 3Ab, bottom) showed abnormalities similar that on OCT. The giant splitting cavity was found in INL at p15. The ONL layer was similar to WT at p15 but rapidly thinned from cell loss from age p21, and only 1 to 3 layers remained by age 6 mo. ONL structure was disrupted by p15, and photoreceptor nuclei were misplaced into the IS and OS regions and the OPL (Fig. 3Ab, bottom, yellow arrows). Both OPL and IS/OS zone narrowed with age and were essentially absent by age 6 mo. GCL ganglion cell layer, IPL inner plexiform layer, INL inner nuclear layer, OPL outer plexiform layer, ONL outer nuclear layer, ILM inner limiting membrane, IS inner segment, OS outer segment, ORRBs outer retinal reflective bands. Scale bars: 200 μm horizontal; 100 μm vertical in OCT images. 50 μm for semi-thin histology sections. Fig. 3Ba shows retinal layer thickness in 8–15 *Rs1h*^−/y^ eyes and 4–8 WT eyes at each age. The outer retina from OPL to RPE shows progressive thinning (left), with reductions of 30% at p21, 34% at p28, 44% at 2 mo, 56% at 3 mo, and 71% at 6 mo (all significant versus WT, unpaired *t* test, *p* < 0.0001). Thickness of the inner retina from ILM to IPL remained normal at each age (right plot). Fig. 3Bb shows ONL and INL cell counts from at least 3 *Rs1h*^−/y^ and age-matched WT controls at each age. ONL cell numbers for *Rs1h*^−/y^ were 250 ± 25 (mean ± SEM), or 29 % reduced at p21; 163 ± 22, or 50% reduced at p28; 92 ± 22, or 64% reduced at 3 mo; 32 ± 25, or 85% reduced at 6 mo (all significant versus WT by Sidak’s multiple comparisons test, *p* ≤ 0.004). The INL had not significantly loss of cells versus WT (Fig. 3Bb, right plot) “(color figure online)”.

The *Rs1h*^*−/y*^ retina was evaluated for morphologic change using OCT imaging (Fig. 3A, a) and H&E stained histology sections (Fig. 3A, b) at six ages (p15 to 6 mo). OCT images of WT retina (Fig. 3A, a, top panel) shows normal organized lamellar structure with defined OCT reflective bands out to 6 mo age, including GCL, IPL, INL, OPL, ONL, and the normal four outer retinal reflective bands (ORRB, i.e., OLM, IS/ep, interdigitation zone and RPE). By comparison, the laminar structure of *Rs1h*^*−/y*^ was disrupted (Fig. 3A, a, second panel) and had large schisis cavities spanning the INL by p15, and by p21 the cavities were already collapsed to only narrow crevices in the INL (3Aa, bottom panel, red arrows). By p28 they continued to reduce further (Fig. 3A, a, bottom panel, green arrows) and disappeared by 2 mo age. The OPL boundary was irregular and the ONL thinner by p21, and the ORRB morphology was distorted and indistinct. ONL thickness continued to diminish rapidly with age. These changes were seen across the retina from center to periphery.

*Rs1h*^*−/y*^ retinal histology showed abnormalities corresponding to those observed on OCT across these age (Fig. 3A, b, bottom). Histology showed giant schisis cavities in INL at p15 (Fig. 3A, b, bottom, left), and the rows of ONL nuclei initially were similar to WT but then thinned rapidly from cell loss beginning by p21. Only 1 to 3 layers of ONL remained by 6 mo age. At p15 the ONL lamination was considerably disrupted, with many photoreceptors misplaced into the IS/OS region and into the OPL (Fig. 3A, b, bottom, yellow arrows). The width of the OPL and IS/OS zones both diminished with age and were essentially absent by 6 mo age. The INL had irregular boarders but retained a normal cell count.

Thickness of the retinal layers was measured on OCT images, and ONL and INL cell numbers were counted on histologic sections from p21 to 6 mo. The effect of age on layer thinning and outer retina cell loss was evaluated (Fig. 3B). Thickness of the outer retina from OPL to RPE was reduced 30% at p21, 34% at p28, 44% at 2 mo, 56% at 3 mo, and 71% at 6 mo, and all changes were significant compared with WT (unpaired *t* test, *p* < 0.0001) (Fig. 3B, a, left plot). The ONL cell count was 29% diminished at p21, 50% at p28, 64% at 3 mo, and 85% at 6 mo, and again all changes were significant between *Rs1h*^−/y^ and WT (Sidak’s multiple comparisons test, *p* ≤ 0.004) (Fig. 3B, b, left plot). No significant difference was noted in inner retinal thickness (Fig. 3B, a, right plot) or for INL cell number of *Rs1h*^*−/y*^ versus WT rats (Fig. 3B, b, right plot).

### Retinal functional status

**Fig. 4:**
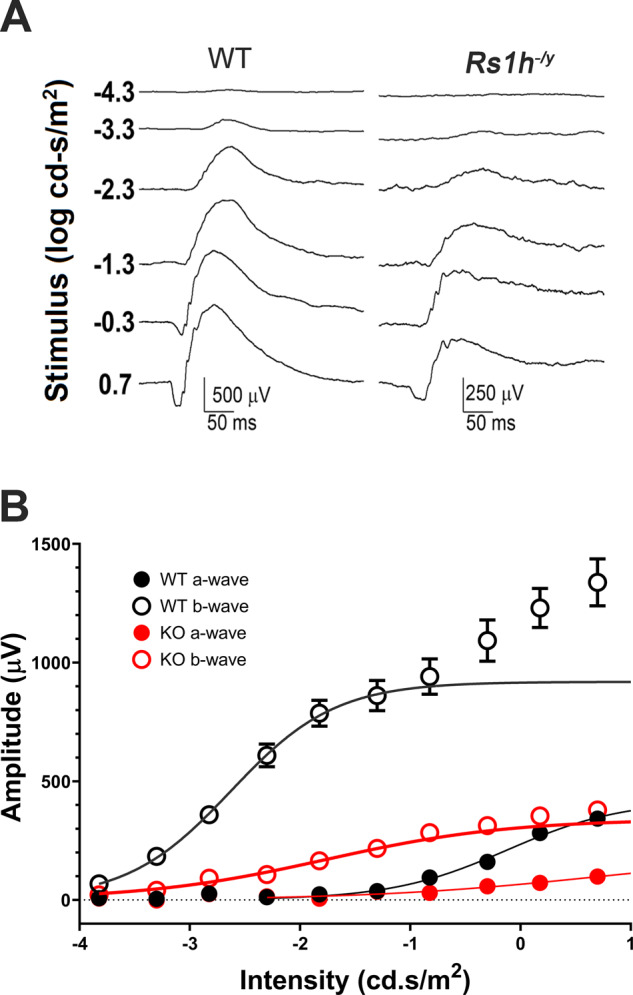
ERG recordings. **A** Representative dark-adapted ERGs of WT and *Rs1h*^−/y^ rats; **B** curve-fitted averaged intensity-response relation for dark-adapted ERG a- and b-waves.

Retinal function was evaluated by ERG recordings at p27-28. Figure 4A shows a series of dark-adapted ERGs of *Rs1h*^−/y^ and WT rats, and averaged intensity-response relations for the a- and b-waves are shown in Fig. 4B. The continuous curves are a fitting of the data by the Naka-Rushton equation. *Rs1h*^−/y^ rats showed diminished a-wave amplitudes with R_max_ of 196 µV versus 421 µV for WT, consistent with the ONL cell loss (Fig 3Ab). The function of b-wave amplitude to stimulus flash luminance has two limbs, with low flash responses up to −0.7 log cd • s/m^2^ dominated by rod inputs, and a separate limb for high intensity flashes which elicits activity of both rods and cones (termed: mixed inputs) [22–24]. Fitting of the Naka-Rushton equation to the low intensity limb yielded R_max_ of 919 µV for WT and 341 µV for *Rs1h*^−/y^, a 63% reduction.

An indication of the input/output relationship for the rod synapse was analyzed using the ratio of R_max_ for the rod-driven b-wave versus the a-wave, and the (b-/a-wave) was seen to be reduced from 2.18 in WT to 1.73 for *Rs1*^−/y^. We also determined the synaptic signaling integrity using b-wave amplitude elicited at −1.8 log cd • s/m^2^ which is purely rod-driven; the a-wave amplitude was elicited at 0.70 log cd • s/m^2^ is also purely rod-driven [22]. This gave a similar result for the averaged (b-/a-wave) ratio of 2.25 ± 0.09 for WT (mean ± SEM, *n* = 7) and 1.71 ± 0.13 for *Rs1h*^−/y^ (mean ± SEM, *n* = 14), which was significant (*p* < 0.05; two-way ANOVA). Under the assumption that the mechanism responsible for generating the b-wave was intact, this change reflects deficient synaptic transmission from rod photoreceptors to the rod bipolar cells [7, 25].

*Rs1h*^−/y^ also showed reduced sensitivity for both the a- and b-waves, indicted by the Naka-Rushton K value. The K value for the *Rs1h*^−/y^ a-wave was 0.70 log cd • s/m^2^ which is considerably higher than the WT a-wave K value of −0.12 log cd • s/m^2^ (0.82 log unit difference). The K value of the *Rs1*^−/y^ b-wave was also considerably higher (−1.76 log cd • s/m^2^) than for WT (−2.63 log cd • s/m^2^), or a 0.87 log unit sensitivity reduction for the XLRS model. This is consistent with reduction of quantum catch in *Rs1h*^−/y^ photoreceptors most likely due to shortened and disorganized ROS [26].

### No deleterious gain-of-function in *Rs1h*^−/+^ heterozygous females with mutant *Rs1h* mRNA

**Fig. 5:**
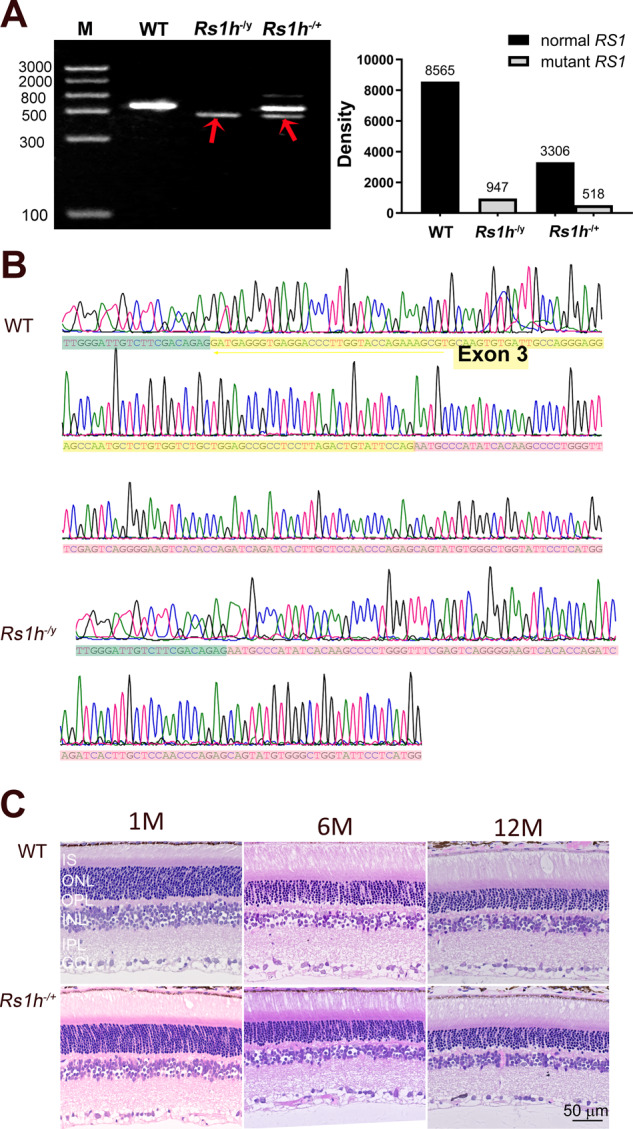
No deleterious gain-of-function effect is detected in heterozygous *Rs1h*^−/+^ females with mutant *Rs1h* mRNA. **A** left, both heterozygous and KO retina shows presence of mutant mRNA by RT-PCR (**A**, red arrows). mRNA band near 600 bp is normal WT *Rs1h;* band near 500 bp is mutant *Rs1h* message of *Rs1h*^−/y^ males and *Rs1h*^−/+^ heterozygous females, both significantly reduced versus WT. **A** right, density (quantity) of mutant *Rs1h* in KO retina was 11% of normal male WT *Rs1h* and ~16% of normal in heterozygous *Rs1h*^−/+^ retina (densities labeled at top of each bar). **B** DNA sequencing of WT and KO RT-PCR products show that *Rs1h* exon 2 (4B, green) connects directly to *Rs1h* exon 4 (5B, pink) with no *Rs1h* exon 3 sequence (5B, top, yellow highlight) in *Rs1h*^−/y^ retina. **C** the morphology *Rs1h*^−/+^ heterozygous retina compares well to WT and without evident difference at ages 1, 6, and 12 mo. GCL ganglion cell layer, IPL inner plexiform layer, INL inner nuclear layer, OPL outer plexiform layer, ONL outer nuclear layer, IS inner segment, OS outer segment. Scale bars: 50 μm “(color figure online)”.

The mutant *Rs1h* mRNA in this model causes a frameshift with PTCs which normally undergoes nonsense-mediated mRNA decay (NMD) to prevent production of harmful truncated protein [27–33]. RT-PCR analysis was performed to determine relative level of mutant *Rs1h* transcription in transgenic rats (Fig. 5). The RT-PCR band density was considerably reduced in *Rs1h*^*−/y*^ retina versus WT, and the density of mutant *Rs1h* band was also significantly lower than normal *Rs1h* in the *Rs1h*^*−/+*^ heterozygous female retina, indicating the majority of mutant *Rs1h* mRNA was eliminated by NMD, as expected. The DNA sequencing analysis indicated that no exon 3 sequences (106 bp) were identified (Fig. 5B, yellow highlighted), and that exon 2 (Fig. 5B, green highlighted) connected directly to exon 4 (Fig. 5B, pink highlighted) without exon 3 in the *Rs1h* mutant retinas (Fig. 5B, yellow highlighted). The DNA sequence analysis confirmed that a small quantity of *Rs1h* mutant mRNA was present in the knockout samples (Fig. 5A, red arrows).

We evaluated for potential toxic effects of truncated protein by close analysis of retinal morphology of *Rs1h*^*−/+*^ heterozygous female retinas at five ages, from 1 to 12 mo (representative histologic morphology is shown with age matched WT, in Fig. 5C). No differences could be detected in comparing mutant to WT retinal lamination, cell placement and layer OCT width of overall organization, including expected number of ONL rows. From this we could detect no evidence of a deleterious gain-of-function effect in the heterozygotes that carry the mutant *Rs1h* mRNA.

### Supplementing *Rs1h*^*−/y*^ retina with AAV8-*RS1* improves retinal morphology

**Fig. 6:**
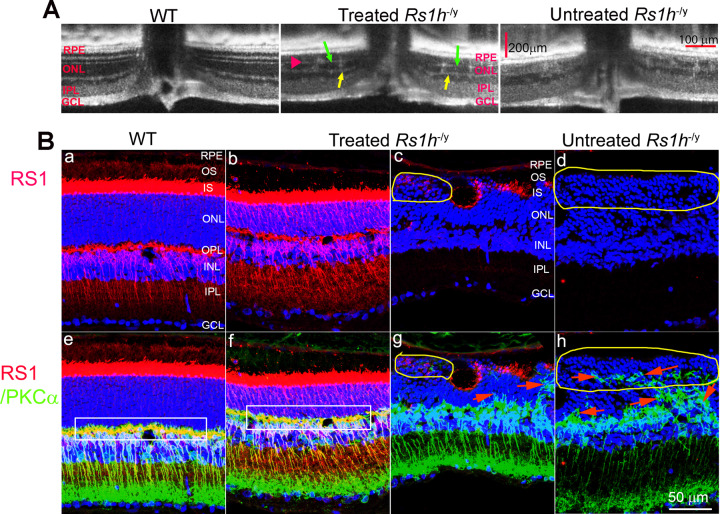
*RS1* gene augmentation gives morphologic improvement of *Rs1h*^−/y^ retina on OCT (Fig. 6A, middle) compared with WT (left) and untreated *Rs1h*^−/y^ retina (right). Treated retina had more organized lamina, with more distinct OPL and ONL margins (yellow arrows). OLM was better defined (green arrows), and subretinal space was preserved (red arrowhead). **B** Shows immunofluorescent retinal images with RS1 antibody (top panel) and RS1/PKCα (red/green) (bottom panel). The images of treated retina were selected from two different regions of one treated rat eye, one with greater RS1 expression (**B** and **F**), and the second with minimal expression (**C** and **G**). The treated *Rs1h*^−/y^ retina with high *RS1* expression has WT RS1 distribution pattern, and organized retinal laminar structure is well organized with all layers preserved. ON-bipolar cells (PKCα label) show an organized synaptic distribution between neurons, as in WT retina (**E** and **F**, white rectangular). By comparison, both the untreated *Rs1h*^−/y^ retina (**D** & **H**) and the low expressing *Rs1h*^−/y^ retina (**C** & **G**) display an interrupted outer retinal structure, with photoreceptors intruding into the subretinal space (**C**, **D**, **G** and **H**, yellow circles) and bipolar cell fibers extending into the ONL and subretinal space (**C**, **D**, **G** and **H**, red arrows). GCL ganglion cell layer, IPL inner plexiform layer, INL inner nuclear layer, OPL outer plexiform layer, ONL outer nuclear layer, IS inner segment, OS outer segment. Scale bars: OCT, 200 μm horizontal and 100 μm vertical; 50 μm for histology sections “(color figure online)”.

We reasoned that if off-target effects or toxic-gain function were responsible for the early, severe and rapid phenotype rather than from Rs1h deficiency, the *Rs1h*^*−/y*^ mutant retina would not benefit from augmentation with normal *RS1*, which had been demonstrated previously for the *Rs1h*^*−/y*^ mouse which gained better retinal morphology and function after gene transfer [1, 7, 10–12]. *RS1* was delivered to *Rs1h*^*−/y*^ eyes at p7-p8 when ONL cell development appeared similar to WT and showed no evident ONL loss (Fig. 6). Twelve weeks after treatment the appearance of the retinal laminar structure was more organized and the OPL and ONL margins were more distinct. On OCT imaging the line representing the OLM reflective band was better defined (6 A, green arrows), and the subretinal space was partially preserved (6 A, red arrowhead). Similar OCT improvements had previously been found for *Rs1h*^*−/y*^ mouse retina after AAV8-*RS1* treatment [12].

Retinal immunofluorescent images of *Rs1h*^*−/y*^ eyes after AAV8-*RS1* treatment are shown in Fig. 6B, one resulting in high *RS1* expression (Fig. 6, b and f) and a second with less *RS1* expression (Fig. 6, c and g), along with an untreated *Rs1h*^*−/y*^ retina (Fig. 6, d & h). The *Rs1h*^*−/y*^ retina with high *RS1* expression showed an expression pattern equivalent to WT, with well organized retinal laminar structure and preservation of all layers as in WT, although the ONL was thinner (Fig. 6a and b). Furthermore, the on-bipolar cells (PKCα labeled) exhibited better organization of photoreceptor synapse to on-bipolar cells (which colocalize with RS1 protein) (Fig. 6e and f, white rectangular).

The *Rs1*^*−/y*^ non-treated eye and the *Rs1*^*−/y*^ retina with only little *RS1* expression both display an interrupted pattern of outer retinal structure, with some photoreceptors misplaced into the subretinal space (Fig. 6. c, d, g and h; yellow circles). Ectopic neurites processes of bipolar cells extend into the ONL layer and the subretinal space (Fig. 6g and h, red arrows). For both the untreated retina and the retina with only low Rs1h expression, the ONL thickness is less than the retina with higher RS1 expression. This indicates that supplementing the *Rs1h*^*−/y*^ retina with normal *RS1* improves the retinal dysmorphology considerably. This pattern of recovery after providing *RS1* at early developmental age is evidence that the degenerative phenotype in *Rs1h*^*−/y*^ eyes was the result of *Rs1h* deficiency and not off-target or toxic-gain function activity.

## Discussion

A rat model of XLRS has advantages over mouse models in eye and body size. Rat genetic retinal dystrophy models were once used widely in medical research until mouse embryonic stem cells (ESCs) became available [34]. The larger body size of rat versus mouse and supports serial blood samples from single animals over time, and this facilitates monitoring humoral immune responses to therapeutic compounds. The rat eye has threefold greater retinal surface area than mouse, and a vitreous cavity is ten-times larger (rat 54.4 μl versus mouse 5.4 μl) [35, 36]. These anatomic advantages make surgical procedures with the rat eye safer and easier, and the larger eye facilitates ocular drug delivery which should increase consistency of result and interpretation than the tiny mouse eye.

The *Rs1h* exon-3-del knockout rat XLRS model showed severe disease phenotype by early age and progressed rapidly compared to the exon 1 deletion *Rs1h*^−/y^ mouse we made previously [1]. This XLRS rat showed generalized disruption of retinal structure, loss of laminar integrity of the OPL and INL, and photoreceptors misplaced into both the OPL and the subretinal space as early as p15. The outer retina of *Rs1h*^*−/y*^ rat was significantly thinner than WT even by age p21, and rapid loss of photoreceptor cells ensued with only 1–3 rows of nuclei remaining by 6 mo age (70–90% ONL cell loss). *Rs1h*-mutant XLRS mouse models are heterogeneous, and this rate of degeneration was observed in the *Rs1h*^−/y^ mice generated by the Weber group in which about half of photoreceptor cells were lost and rod outer segment (ROS) was nearly completely lost by 2 mo age [2]. The *Rs1h*^−/y^ rat had remarkably large cavities at p15, similar to the *Rs1h*^−/y^ mice created by Liu et al., which included replacing exon 1-3 with LacZ reporter gene one *Rs1h*^*−/y*^ mouse model, and two knock-in mouse models carrying either a C59S or an R141C point mutant substitution [3]. Except for the exon 1 deletion *Rs1h*^−/y^ mouse we made previously, the phenotype onset and progression of this rat model roughly follows that of the faster of the current mouse models (Table 1) [2, 3, 37, 38]. What appears different in this rat model is the rapid emergence and collapse of the schisis cavities. These form over only a few days from P8 to p15, and they then diminish dramatically in only 1–2 weeks, which is different from the mouse models. The basis for the range of phenotype remains unclear, but heterogeneity is also found for human XLRS, both across and within family genotypes [39–41].

**Table 1:** Phenotype comparison of XLRS rat to XLRS mouse models.

	*Rs1h*	Phenotype		Schisis cavities		Photoreceptor^a^			ERG	Reference
	target	Age of	Age of disruted	Age of	Age nearly	Age of 50%	Age of 80%	Age ROS severely	Dark-adapted	
	region	first seen	lamelar structure	maximum	resolved	ONL loss	ONL loss	shortened	response	
Rat	E3 deletion	p8 to p15	p15	p15	1 M	1 M	8 M	5–6 M	a & b-waves reduced b-wave loss > a-wave	Current paper
Mouse	E3 deletion	p18	No data	2 M	No data	1–2 M	7 M	2 M	a & b-waves reduced b-wave loss > a-wave	Ref. # [2, 37]
Mouse	Initiation + E1 deleted	p18	p21	3-4 M	8-10 M	8 M	12 M	16 M	a & b-waves reduced b-wave loss > a-wave	Ref. # [38]
Mouse	E1-3 deletion	p15^a^	p15	p15	after 4 M	2–3 M	No data	No data	a & b-waves reduced b-wave loss > a-wave	Ref. # [3]
Mouse	C59S substitution	p15^a^	p15	p18	after 4 M	2–3 M	No data	No data	a & b-waves reduced b-wave loss > a-wave	Ref. # [3]
Mouse	R141C substitution	p15^a^	p15	p15	after 4 M	2–3 M	No data	No data	a & b-waves reduced b-wave loss > a-wave	Ref. # [3]

*ONL* Outer Nuclear Layer, *ROS* rod outer segments.

^a^The earliest time checked.

The rod-driven dark-adapted ERG a-wave was impaired even by young age, with R_max_ reduced by about 50% at P28 (421 µV for WT, versus 196 µV for *Rs1h*^*−/y*^). This reduction can be largely accounted for by the photoreceptor loss of ~50% ONL cell reduction by p28 (Fig. 3Bb). Previous quantitative study of the mutant P23H rhodopsin transgenic rat showed progressive loss of a-wave sensitivity tracking rod photoreceptor loss, while b-wave responses were relatively less affected due to trans-synaptic compensation such that the relatively larger b-wave actually which increased the (b-/a-wave) ratio [42]. In contrast, the *Rs1h*^*−/y*^ rat showed proportional reductions of both the a- and b-wave sensitivities (~0.8 log unit, Fig. 3), indicating that rod synaptic transmission to rod bipolar cells also appears to be impaired by the *Rs1h* mutation. We previously found a reduction of (b-/a-wave) ratio for the *Rs1h*^*−/y*^ exon-1-del mouse [43]. The reduced ratio is also found for many human XLRS individuals.

The *Rs1h*^*−/y*^ exon-3-del rat phenotype appears more severe at early age than most XLRS patients, less frequently show much reduction of the photoreceptor ERG a-wave across the lifespan [44]. Before concluding that the phenotype is due to Rs1h deficiency, we screened for off-target CRISPR-Cas9 effects, but none were identified. Off-target effects are diluted by mating with WT as the phenotype severity will become milder successive generations [45, 46]. However, the severity of *Rs1h*^*−/y*^ pathology remained consistent across generations 1–4, nor did the heterozygous females exhibit retinal dysmorphology which off-target effects would cause. In addition, the size, weight, appearance, reproductivity and lifespan of the *Rs1h*^*−/y*^ rats are comparable to WT.

While nonsense medicated decay would quickly degrade most mRNA that contains PTCs [27, 30, 31, 33], a small proportion of aberrant *Rs1h* mRNA was detected in both *Rs1h*^*−/y*^ and heterozygous *Rs1h*^*−/+*^ retinas. However, abnormal morphology was not found in heterozygous *Rs1h*^*−/+*^ females, and we conclude that mutant *Rs1h* mRNA does not itself cause disease in this rat model. This is consistent with the *Rs1h*^*−/y*^ mouse created by Liu et al. [3], and it is also constant with lack of clinical findings for human female *RS1*^*−/+*^ carriers who with rare exception do not show retinal pathology despite carrying an *RS1* nonsense mutant allele [47–49]. Additional supporting evidence comes from supplementing the *Rs1h*^*−/y*^ retina with a normal *RS1* gene via AAV8-*RS1* delivery, as this helped preserve retinal laminar structure in the *Rs1h*^*−/y*^ male retina and partial preserved reduced the photoreceptor loss. On these bases, we propose that the XLRS rat phenotype results from lack of normal RS1 protein and is a suitable model of an Rs1h-deficient animal.

This *Rs1h*^*−/y*^ rat XLRS model shares disease features with human XLRS, including the development of schisis cavities within OPL and INL, degeneration of photoreceptors with age although, and a relative reduction in ERG b-wave to a-wave amplitudes. While this rat model does not show a “negative ERG waveform,” this occurs in about half of human XLRS cases, and the reduced (b-/a-wave) ratio in this rat, as in many XLRS humans, is consistent with impaired synaptic transmission from rod photoreceptors to their bipolar cells [7, 38]. Administering the AAV8-*RS1* vector at age p7-p8 shows the presence of a therapeutic window in this rat model to monitor treatment outcome, but it will be most useful at young age before structural loss of photoreceptors. We do not yet know whether the positive outcome with AAV8-*RS1* results from preservation of cells or a reduction in loss, or both.
